# Research on Fatigue–Healing Performance of Asphalt Mixture Based on the Semicircular Bending Test

**DOI:** 10.3390/ma16196382

**Published:** 2023-09-24

**Authors:** Lijun Wang, Peifeng Cheng, Qiang Zhao

**Affiliations:** School of Civil Engineering and Transportation, Northeast Forestry University, Harbin 150040, China; chengpeifeng@nefu.edu.cn (P.C.); brzq@nefu.edu.cn (Q.Z.)

**Keywords:** road engineering, fatigue–healing, semicircular bending fatigue test, gray relational analysis, asphalt mixture

## Abstract

In order to study the self-healing performance of macroscopic fractures of asphalt mixtures, semicircular bending (SCB) tests were used to test 90# base asphalt mixtures, SBS (Styrene–Butadiene–Styrene) modified asphalt mixtures, and SBS + CR (Chloroprene Rubber) composite modified asphalt mixtures. The F-H-F (the asphalt mixture specimen was fatigued for a certain number of times, then healed under certain conditions, and then fatigued until destroyed) test was carried out, and the fatigue life recovery rate of the fatigue test before and after healing was defined as the healing index (HI). The gray correlation analysis method was used to judge the influence degree of influencing factors on fatigue–healing according to the correlation index. The results show the type of asphalt has the most significant influence on the healing ability of the asphalt mixture. In the case of complete healing, the fatigue–healing performance of the SBS + CR composite modified asphalt mixture was the best, followed by the SBS-modified asphalt mixture, and 90# base asphalt. When the healing temperature is close to the softening point of asphalt, the healing performance of 90# base asphalt is better when the healing temperature is low. When the healing time is longer, the healing performance is better, and there is an optimal healing time. The healing index decreased with the increase in the degree of damage. When the degree of damage is too large, the asphalt mixture will be difficult to heal.

## 1. Introduction

As a material with viscoelastic properties, the asphalt mixture is damaged to a certain degree, and given a certain degree of maintenance, its various mechanical properties can be restored. This phenomenon is known as asphalt self-healing. This phenomenon was first discovered by Bazin et al. [1]. He respliced the damaged trabeculae and maintained them at different temperatures. The self-healing properties of asphalt mixtures have been demonstrated for the first time and have also attracted widespread attention from researchers.

Qiu [2] and other scholars researched macroscopic analysis and studied the effects of healing time and temperature on the healing behavior of asphalt mixtures through the damage–healing–damage test method. Scholars such as Bommavaram [3] studied the fatigue performance changes of asphalt mixture during fatigue loading. They found that repeated loading will cause microcracks inside the specimen, and the fatigue performance will gradually decrease. However, the fatigue performance of the mixture will be improved after proper maintenance. Little [4] found through experiments that the aging effect will reduce the healing performance of the asphalt mixture. Garcia [5] found through research that too high a curing temperature will reduce the bonding between asphalt and aggregates and the ability of the mixture to resist deformation, leading to a decrease in its self-healing performance. Chinese scholars Huang Weidong et al. [6] used the N_fNM_ method to study the influence of different asphalt dosages and curing temperatures on the fatigue self-healing of SBS-modified asphalt mixture. He found that prolonged curing can improve its self-healing performance. In addition, he also proposed a linear equation considering factors such as fatigue performance after healing, the loading level, and the amount of asphalt. He Fan et al. [7] used the fracture–heal–re-fracture test of a three-point bending beam, introduced the strength recovery rate as the self-healing index, and studied the self-healing properties of three kinds of asphalt mortars under different healing times and temperature conditions. Sun Daquan et al. [8] studied the effects of SBS, rock asphalt, and surface active modifiers on asphalt self-healing ability through dynamic shear rheometer. Lin Peng et al. [6] used the single-factor comparative analysis method to study three influencing factors on the fatigue self-healing ability of SBS-modified asphalt mixture through a four-point bending fatigue test. Tang Wen [9] and others studied the influencing factors and evaluation methods of asphalt self-healing behavior by introducing the self-healing rate (healing rate, referred to as HR) and self-healing rate (healing index, referred to as HI).

It is in line with the concept of green development in China to recognize the main influencing factors on the healing of asphalt mixture and to use the self-healing performance of asphalt mixture to maintain the pavement before macroscopic damage occurs. Using the self-healing properties of asphalt mixture to make the damaged pavement self-heal and prolong the service life of the road is also a new hotspot in future research.

The four-point bending fatigue test of small beams is usually used for research on the fatigue performance of asphalt mixture [10]. However, in recent years, researchers throughout the world have gradually realized that when using the four-point bending fatigue test to evaluate the performance of the built asphalt pavement, the simulation effect on the thinner asphalt pavement is better. However, the actual stress state of the pavement structure with a thick asphalt mixture layer is different from the pure bending stress state of the girder specimen, which leads to some problems when using the test results adopted in the design standard to evaluate the performance of the pavement structure layer. Compared with the four-point bending fatigue test, the semicircular bending (SCB) test is simple to operate, has a wide range of specimen sources, and has good repeatability [11,12]. It is a new method for evaluating the cracking performance of asphalt mixtures in recent years and has gradually attracted the attention of researchers at home and abroad [13,14]. Fu Xin [15] used ANSYS to analyze the test parameters of SCB with notches and found that the stress of the semicircular specimen was closer to the stress state of the asphalt pavement structure. Liu Yu et al. [16] used the SCB test to evaluate the strength of the asphalt mixture and found that the SCB test is more suitable for evaluating the tensile performance of the asphalt mixture. Wu Fan [17] used the semicircular bending fatigue test combined with DIC digital elastic correlation technology to study the influence of slits on the fatigue performance of the asphalt mixture. It established a fatigue equation in the form of a power function. Liu Hailin [18] studied the effect of fiber on the self-healing performance of asphalt mixture through a semicircular bending fatigue test and found that fiber positively improves the healing index of the asphalt mixture.

The literature shows that the previous studies focus more on the evaluation of four-point bending fatigue self-healing of asphalt mixture. The study is also more inclined to study the comparison of tensile strength of asphalt mixture before and after self-healing. The influence of multiple factors on fatigue self-healing in semicircular bending of asphalt mixture is seldom studied. Therefore, this study focuses on evaluating the influencing factors of fatigue self-healing in semicircular bending. 90# base asphalt and SBS-modified asphalt are commonly used asphalt in northern China at present. The reuse of waste rubber powder is beneficial to protect the environment, and the incorporation of rubber powder can improve the performance of modified asphalt. Therefore, 90# base asphalt, SBS-modified asphalt and SBS + CR composite modified asphalt are selected for research in this paper.

In this paper, the semicircular bending fatigue–healing–fatigue test of asphalt mixture is used to analyze the healing performance of asphalt mixture through the change of fatigue life before and after healing. At the same time, the effects of healing temperature, healing time, degree of damage, and interval time on the self-healing performance of 90# base asphalt mixture, SBS-modified asphalt mixture, and SBS + CR composite modified asphalt mixture and the differences among the three were studied, provide a reference for the fatigue–healing technology of asphalt pavement.

## 2. Materials and Methods

### 2.1. Raw Material

Number 90# base asphalt, SBS star modifier, and 40 mesh tire rubber powder (CR) were used. SBS-modified asphalt and SBS + CR composite modified asphalt were prepared using a high-speed shearing machine with an agitator. The preparation method of SBS-modified asphalt is as follows: 90# base asphalt, 4.5% (mass fraction) SBS modifier and 0.15% stabilizer are cut for 30 min in a 6000 r/min shear machine at 180 °C. Then, stir at a rate of 250 r/min for 60 min. Finally, the SBS-modified asphalt was obtained by swelling in the oven at 160 °C for 30 min. The preparation of SBS + CR composite modified asphalt is as follows: 4.5% SBS modifier, 0.15% stabilizer, and 10% CR are uniformly mixed into 90# base asphalt at 180 °C. Cut for 35 min in a high-speed shear at 6000 r/min and stir for 60 min at 170 °C. Finally, the SBS + CR composite modified asphalt was obtained by swelling in the oven at 170 °C for 60 min. Table 1 shows the primary indicators of the three kinds of asphalt. Asphalt performance meets the requirements of “Technical Specifications for Construction of Highway Asphalt Pavements” (TG F40-2004; Technical Specifications for Construction of Highway Asphalt. China Communications Press: Beijing, China, 2005). The coarse and fine aggregates are all basalt, and the filler is limestone. The performance meets the requirements of the “Test Methods of Aggregate for Highway Engineering” (JTG E42-2005; Test Methods of Aggregate for Highway Engineering. China Communications Press: Beijing, China, 2005).

The AC-16 gradation type commonly used in the northern black pavement was selected. In order to be closer to engineering practice, the study uses the median value of the mineral material gradation range as the synthetic gradation according to the “Technical Specifications for Highway Asphalt Pavement Construction” (JTGF40-2004). The design grading is shown in Table 2.

Through the Marshall mix ratio design method, it is determined that the optimum asphalt ratio of AC-16 base asphalt mixture is 4.5%; the ratio of asphalt mixture of SBS-modified asphalt mixture and SBS + CR composite modified asphalt mixture is the same, both being 4.7%; the porosity, namely air voids, of the asphalt mixture is controlled within 4.0 ± 0.5%.

### 2.2. Test Specimen Production

**Figure 1:**
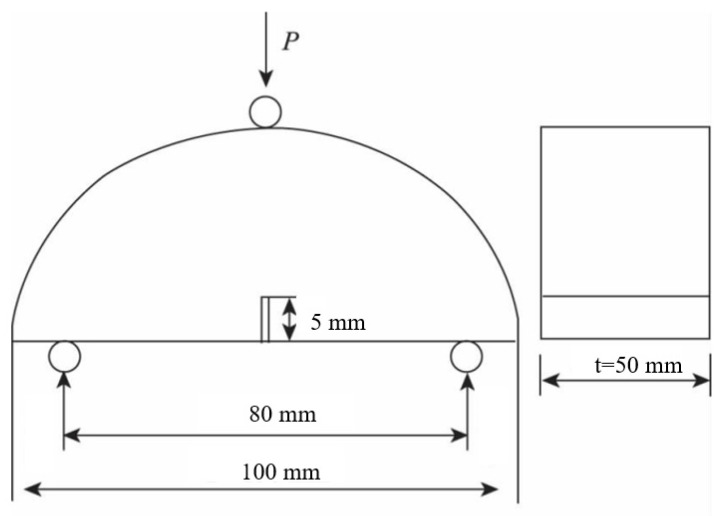
Dimensions of semicircular bending specimen.

The study found that when the thickness of the semicircular specimen is 50 mm, its mechanical response tends to be stable [19,20]. Therefore, the thickness of the semicircular specimen is selected as 50 mm in this study. A cylindrical specimen with a diameter of 100 mm and a height of 180 mm was formed by the rotary compaction method proposed by Superpave. A high-precision double-sided saw was used to cut off the 15 mm part of the asphalt mixture at both ends of the cylindrical specimen and cut the remaining part to obtain three cylindrical specimens with a height of 50 mm and flat top and bottom surfaces. Finally, the cylindrical specimen with a height of 50 mm is cut into two semicircular specimens along the diameter, and the cutting allowable deviation is controlled within 2 mm. A slit with a depth of 5 mm is cut on the centerline of the vertical diameter direction at the bottom of the semicircular specimen to ensure that the specimen is cracked from the middle during the test. The dimensions of the semicircular specimens are shown in Figure 1.

### 2.3. Fatigue Test

#### 2.3.1. SCB Test

**Figure 2:**
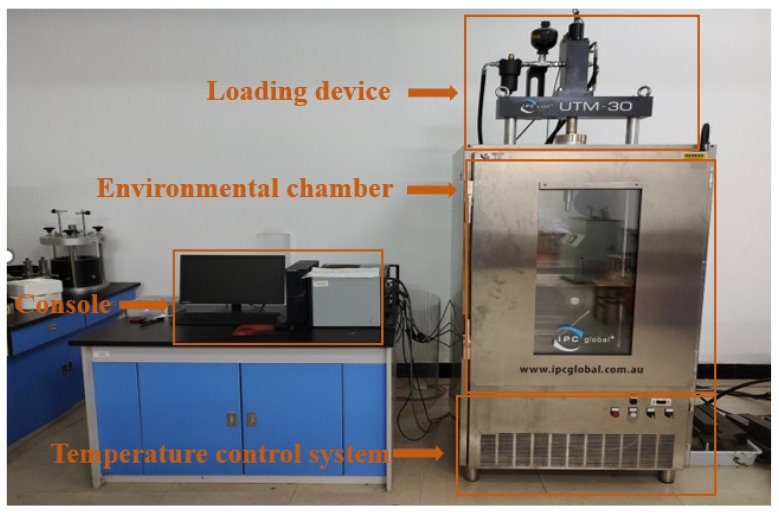
UTM-30 Multifunctional Asphalt Mixture Testing System.

**Figure 3:**
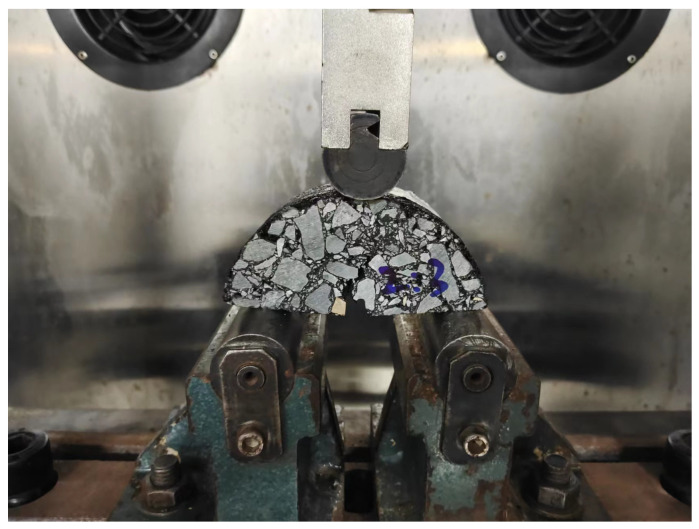
Test specimen under load.

**Figure 4:**
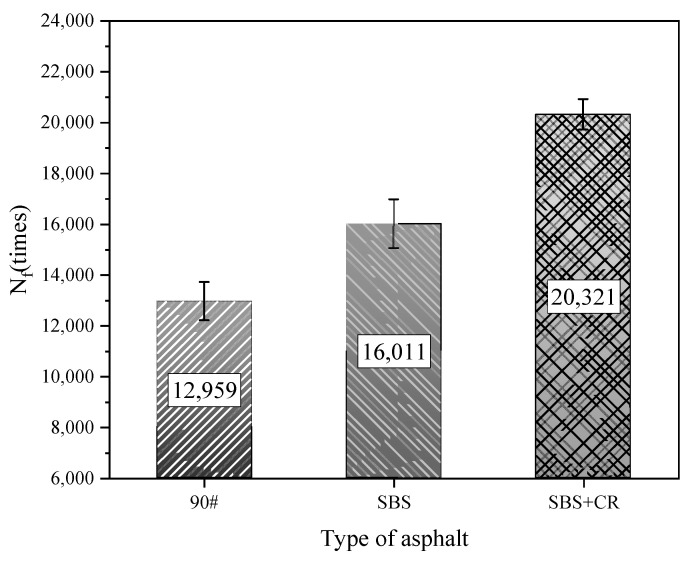
Fatigue life Nf of asphalt mixture.

The IPC Global UTM-30 servo tester is used to carry out the fatigue loading test on the semicircular specimen, as shown in Figure 2. The distance between the two fulcrums of the test is 80 mm, the test temperature is 15 °C, the loading frequency is 10 Hz, the stress control mode is adopted, and the stress ratio is 0.3. The load acts on the top of the semicircular specimen at the same straight line as the bottom notch, using half and half sine wave loading, the test is terminated when the specimen has failed, and the number of loadings is recorded as the fatigue life of the mixture (Nf). Each group has four parallels. The specimen’s fatigue life is taken as the average value after removing the error. The specimen under loading is shown in Figure 3. The fatigue life of the three asphalt mixtures is shown in Figure 4. “90#” in the article represents 90# base asphalt mixture (90# represents the same meaning below)

It can be seen from Figure 4 that the fatigue performance of base asphalt is the worst among the three kinds of asphalt mixtures, and its fatigue life is only 12,959 times; the fatigue performance of SBS-modified asphalt mixture is excellent, and its fatigue life is 16,011 times; the fatigue performance of SBS + CR composite modified asphalt mixture is the best, and the fatigue life is 20,321 times.

#### 2.3.2. Healing Index

The study refers to the specimen subjected to the fatigue test for the first time as a new specimen. New specimens were healed after fatigue testing. The specific process is to place the specimen in a constant temperature oven with a set healing temperature (such as 60 °C, etc.), keep it warm for a set healing time (such as 2 h, 4 h, etc.) without applying an external load, and then cool it to room temperature. After the healing treatment, the semicircular specimen subjected to the fatigue test for the second time is called the healed specimen, and the fatigue life of the healed specimen is recorded as *N_fH_*.

The fatigue–healing index after healing was used as the evaluation index of the healing performance of the mixture. The fatigue life of the mixture is calculated by subtracting the remaining fatigue life if it is not healed from the fatigue life after healing, and then comparing it with the preset damage fatigue times, which is the fatigue–healing index of the mixture. The calculation formula is as formula 1.

(1) $$HI=\frac{N_{fH}-(1-r)N_{f}}{r\times N_{f}}\times 100\%$$

Where *HI* is the fatigue–healing index; *N_fH_* is the fatigue life of the specimen after healing; *N_f_* is the fatigue life of the undamaged specimen tested above; *r* is the preset degree of damage.

## 3. Results

### 3.1. Effect of Temperature on Fatigue–Healing

Wu et al. [21] defined healing as when two identical materials are above the glass transition temperature, the contact interface gradually disappears, and the crack healing caused by molecular diffusion at the interface will increase the mechanical strength at the interface. The definition explicitly points out one of the essential conditions for healing—temperature, which must be higher than the material’s glass transition temperature. Since asphalt is a viscoelastic material, an increase in temperature causes it to soften and flow. Asphalt has not aged within a specific temperature range, and the flow range is limited. The higher the external temperature, the greater the activity of asphalt molecules and the stronger the diffusion ability, which has a specific impact on the filling of the surface and cracks of the bonded aggregate. Therefore, it enhances the self-healing ability of the asphalt mixture. However, the asphalt is close to Newtonian fluid when the temperature rises to a particular stage. Healing occurs, and the temperature rise again has little effect on the healing of the mixture and may even produce adverse reactions.

**Figure 5:**
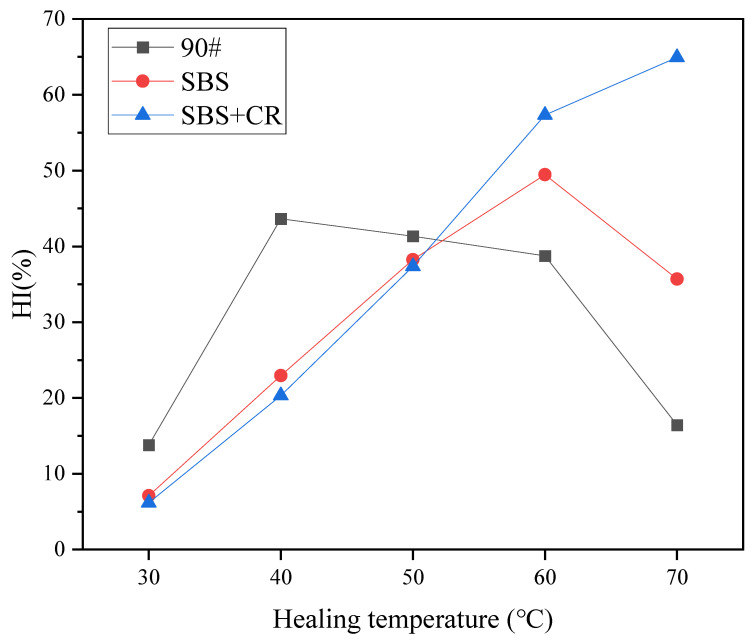
Curve of healing index of asphalt mixture with healing temperature.

The five different healing temperatures (30 °C, 40 °C, 50 °C, 60 °C and 70 °C) were used, taking AC-16 asphalt mixture as the research object, through the semicircle bending fatigue test, three kinds of asphalt mixture (90#, SBS and SBS + CR) for F-H-F tests. The healing time was 6 h, and the degree of damage was 50%. The specific test results are shown in Figure 5.

It can be seen from Figure 4 that after healing in a specific environment, the fatigue life healing index of 90# base asphalt mixture with a degree of damage of 50% can reach up to 43.6%; the fatigue life healing index of SBS-modified asphalt mixture can reach up to 49.5%; and the fatigue life healing index of asphalt mixture modified with rubber powder can reach up to 64.9%. When the healing temperature of the three kinds of asphalt mixture is lower than its softening point, the healing index increases with the increase in the healing temperature. The healing index of 90# base asphalt and SBS-modified asphalt mixture has the maximum value with temperature change, while the softening point of SBS + CR composite modified asphalt is 72 °C, which is higher than the maximum value of the healing temperature set in the test (70 °C). Therefore, at the temperature set by the test conditions, the healing index increases with the increase in the healing temperature, and the growth rate of the healing index slows down when the temperature is close to the softening point, indicating that 70 °C is also close to the maximum healing temperature. It can be seen that there may be an optimal healing temperature for the three kinds of asphalt mixtures, and the healing temperature is lower than the softening point. When the healing temperature is higher than the optimal healing temperature, the improvement effect on the healing performance of the asphalt mixture is not good and may even have a negative effect. When the healing temperature is too high, the viscosity of asphalt decreases and the flow ability increases. The bitumen in the crack is lost and the specimen cannot be repaired well.

The three kinds of asphalt mixtures exhibit different healing properties at different temperatures, and this difference can also be analyzed from the healing mechanism of the three kinds of asphalt. The healing mechanism of the 90# base asphalt mixture is mainly the viscous flow of asphalt into the crack and the mutual wetting and diffusion of asphalt molecules at the crack interface. The higher the temperature, the greater the asphalt flow and molecular diffusion rates. When the temperature is close to the softening point, the asphalt is close to the fluid state and has good fluidity, promoting the healing of cracks. Moreover, 90# base asphalt has a lower softening point, showing higher healing at lower temperatures. The self-healing mechanism of the modified asphalt mixture is related to the viscous flow and diffusion of the asphalt matrix and the polymer properties. SBS polymers are styrene–butadiene–styrene triblock copolymers. Typically, polystyrene gathers together to form microdomains and disperses between polybutadiene continuous phases, which play the role of physical crosslinking, fixed chain segments, and vulcanization enhancement so that SBS-modified asphalt has good elasticity. The healing of the SBS-modified asphalt mixture results from the joint action of molecular flow and diffusion of the asphalt matrix and elastic recovery of SBS segments. Similarly, CR plays a different role in the healing of SBS + CR composite modified asphalt mixture. After the rubber powder is mixed with asphalt, chemical modification, physical dissolution, and swelling occur. Rubber powder changes the structure of asphalt to a certain extent, thus significantly affecting the performance of asphalt. The healing of composite modified asphalt mixture has the elastic recovery effect of rubber powder based on SBS-modified asphalt.

### 3.2. Effect of Healing Time on Fatigue–Healing

Healing time largely influences the healing properties of a mixture. Studies by many scholars have shown that, in general, the longer the loading interval, the better the healing effect of the mixture [22,23].

**Figure 6:**
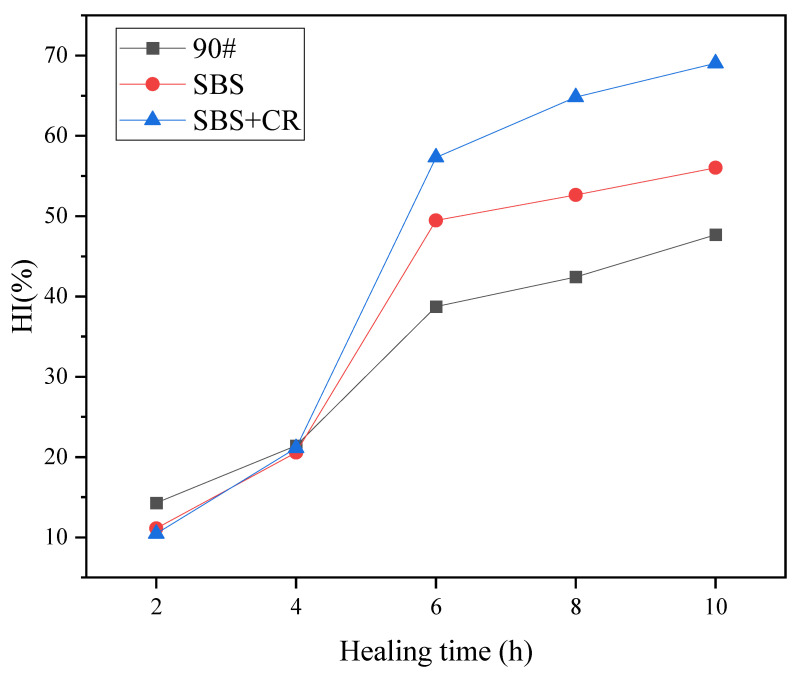
Curve of healing index of asphalt mixture with healing time.

Five different healing times (2 h, 4 h, 6 h, 8 h, and 10 h) were used, the healing temperature was 60 °C, and the degree of injury was 50%. The AC-16 asphalt mixture was selected as the research object, and the F-H-F test of three kinds of asphalt mixtures (90#, SBS, and SBS + CR) was carried out by semicircular bending fatigue test—the healing index changes with the healing time, as shown in Figure 6.

In general, the healing index increases with increasing healing time, but the specific trend varies with the type of mixture and the healing index. It can be seen from Figure 6 that the HI values of the three asphalt mixtures increase with the increase in the healing time. When the healing time is short, the 90# base asphalt mixture has the best healing performance; followed by the SBS-modified asphalt mixture; with the healing performance of the SBS and rubber powder composite modified asphalt mixture being the worst. With the increased healing time, the healing performance of the SBS and rubber powder composite modified asphalt mixture improved continuously, surpassing the SBS-modified asphalt mixture and 90# base asphalt mixture to become the best. On the contrary, the healing performance of the 90# base asphalt mixture gradually lags with the increase in healing time and, finally, is ultimately lower than the other two asphalt mixtures. The reason is that the softening point of the base asphalt is low, and the base asphalt can wet, flow, and spread better in a shorter time. Therefore, the base asphalt heals better with a shorter healing time. As the healing time increases, the fully wetted and flowing modified asphalt mixture better exhibits its excellent healing ability, so the healing performance of the modified asphalt mixture with a long healing time catches up with and exceeds that of the base asphalt.

Compared with the healing time of 4 h, the HI values of the three kinds of asphalt mixtures cured after 6 h increased by at least 17.3%, and the highest increased by 36.2%; after 8 h and 10 h, the HI values of the three kinds of asphalt mixtures increased by only 7.5%. This shows that although the HI value increases with the healing time, there is also a most cost-effective healing time. When that time is exceeded, the growth rate of the HI value of the asphalt mixture decreases rapidly, and continuing to prolong the healing time has a significant impact on the healing process. The improvement in ability is not apparent.

### 3.3. Effect of Injury Degree on Fatigue–Healing

**Figure 7:**
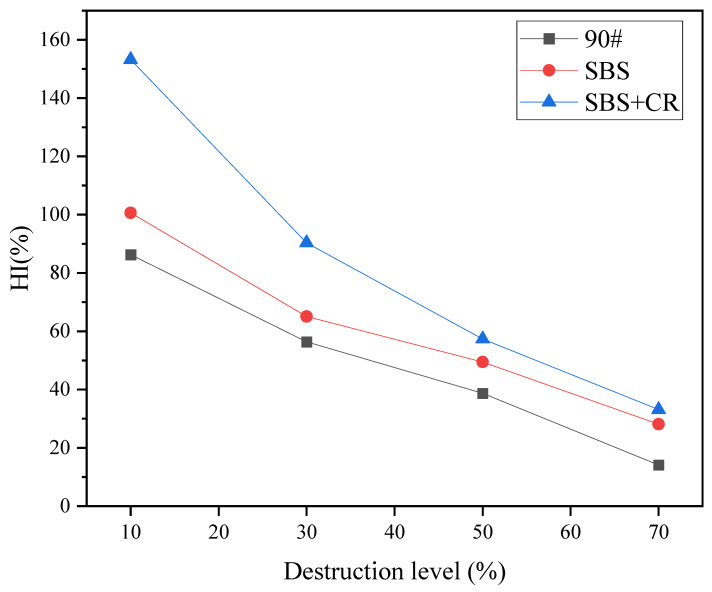
Curve of healing index of asphalt mixture with degree of damage.

The degree of pavement damage determines the degree of pavement maintenance and repair. Similarly, the degree of damage of the asphalt mixture also greatly influences fatigue–healing. F-H-F test, the change of healing index with the degree of injury, is shown in Figure 7.

It can be seen from Figure 7 that when the damage level is low (10%), the healing index HI values of SBS-modified asphalt mixture and SBS + CR composite modified asphalt mixture reached 100.6% and 153.2%, indicating that after healing, The fatigue life is higher than that under normal conditions. It can be seen that the semicircular bending specimen has been damaged by 10% of the fatigue life before healing. Hence, the fatigue performance of the two mixtures after complete curing is better than before the damage, that is, the “forging effect” occurred. The healing action does not entirely cause this phenomenon. According to the research of previous scholars [24,25], fatigue loading has two effects on materials: (1) When the number of loading cycles is small, the internal structure of the material is rebuilt under the action of external loads, and even some structural defects are repaired. At this time, the material structure is equivalent to being strengthened, and the fatigue life will be improved; (2) When the number of loading reaches a specific critical value, the internal structure of the material is gradually destroyed, and cracks appear inside. With the increase in loading times, the fatigue performance of the material decreases gradually and, finally, cracks and fails. Analyzing the “forging effect” of this test, a small amount of fatigue loading changes the internal structure of the asphalt mixture specimen, combined with the wetting and diffusion of the asphalt during the healing process. Repairing structural defects bridges some of the specimen’s internal pores, making the specimen’s fatigue performance recover to the original or even improve. Compared with the other two asphalt mixtures, the fatigue resistance of 90# base asphalt mixture is relatively poor. At the same damage level, the fatigue loading times of 90# base asphalt mixture specimen exceeded the critical value required for the forging effect. As a result, its fatigue performance cannot be restored to the initial value.

Figure 7 shows that as the degree of damage increases, the HI value of the asphalt mixture healing index gradually decreases. This is because as the number of loads increases, the internal cracks in the asphalt mixture gradually develop and expand, and the test healing cannot wholly bridge the cracks, so the healing index of the asphalt mixture specimens gradually decreases. Under different damage levels, the healing rate of the base asphalt mixture was significantly lower than that of the other two mixtures. Analysis of the reasons shows that the viscoelasticity of base asphalt is low, and its ability to inhibit crack development is poor, making the mixture’s internal cracking degree relatively large under the same damage level. Ultimately, it is more difficult to repair cracks with asphalt.

### 3.4. Effect of Interval Time on Fatigue–Healing

**Figure 8:**
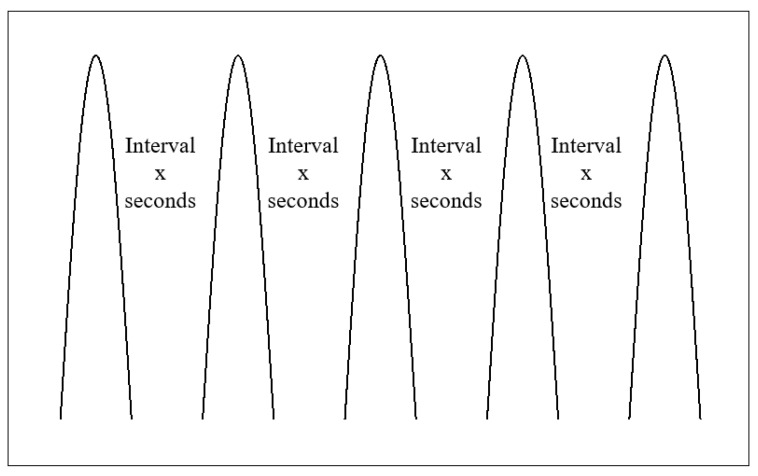
Schematic diagram of interval loading waveform.

In the actual road traffic situation, the vehicle load on the road surface is not continuous, but the traffic load with intervals. Speed, spacing between vehicles, wheel-axle combination, and traffic density all contribute to load discontinuity. Most indoor asphalt mixture fatigue studies adopt the continuous and uninterrupted dynamic cyclic loading mode, which does not conform to the actual road traffic load conditions. Domestic and foreign studies on the fatigue self-healing behavior of asphalt and mixtures mainly introduce a rest period in the fatigue test. They analyze their self-healing ability by comparing the fatigue behavior of asphalt and mixtures with and without a rest period. There are mainly two ways to introduce the rest period:

(1) There is a rest interval through continuous loading of a certain proportion of the fatigue life of the load, the loading is performed after a certain period, and then the loading is repeated until the specimen is entirely fatigued;
(2) In interval loading, a rest period is introduced in each loading cycle, and the rest period is short. The schematic diagram of the waveform is shown in Figure 8.

Studies by scholars have shown that [26,27] the intermittent loading method is more in line with the traffic conditions on the road surface. The intermittent loading corresponds to the actual force on the asphalt pavement when the continuous vehicle load is applied. The time interval is between the passing of the vehicle’s front wheels and the vehicle’s rear wheels. Existing studies have shown that the impact of the intermittent time on the fatigue life of asphalt and asphalt mortar mainly occurs within the first 1 s before the load is removed. In addition, considering the long test period of the fatigue test itself, the test period will be significantly increased after introducing the intermittent period. Therefore, this experiment’s maximum loading interval time is set to 1 s. A total of 5 groups of tests are set up, namely the fatigue test with intermittent 0 s, that is, no intermittent fatigue test, and the fatigue test with intermittent time of 0.1 s, 0.2 s, 0.5 s, and 1 s, respectively.

#### 3.4.1. Fatigue Life under Spaced Loading

**Figure 9:**
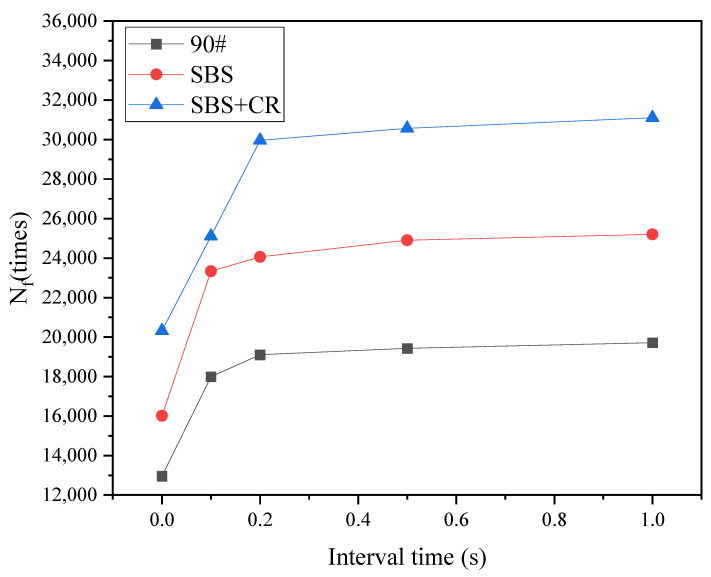
Fatigue life changes with interval time.

Interval loading significantly affects the fatigue life of asphalt mixtures [17]. The fatigue life must be obtained first to study the effect of interval loading on fatigue–healing. Semicircular bending fatigue tests were carried out on the asphalt mixture specimens, and the intermittent times were 0.1 s, 0.2 s, 0.5 s, and 1 s. The test results are shown in Figure 9.

It can be seen from Figure 9 that the fatigue life of the asphalt mixture under interval loading is significantly enhanced. When the interval time increases from 0 to 0.1 s, the asphalt mixture’s fatigue life and growth rate increase rapidly. The interval time of 0.1 s makes fatigue life increase by 38.84%. When the intermittent time increases from 0.1 s to 0.2 s, the fatigue life and the growth rate of fatigue life still increase accordingly; when the load intermittent time gradually increases from 0.2 s to 1 s, the fatigue life and the growth rate of fatigue life of asphalt mixture keep increasing trend slowly, the growth rate was significantly lower than the previous stage. According to the test data, it can be concluded that the impact of the interval loading interval time on the fatigue life of asphalt mixture mainly occurs within the first 0.2 s before the load is removed, especially the most significant within the first 0.1 s after the load is removed. Prolonging the intermittent time after the intermittent time exceeds 0.2 s has little effect on the fatigue life of the asphalt mixture.

#### 3.4.2. Effect of Spaced Loading on Healing

**Figure 10:**
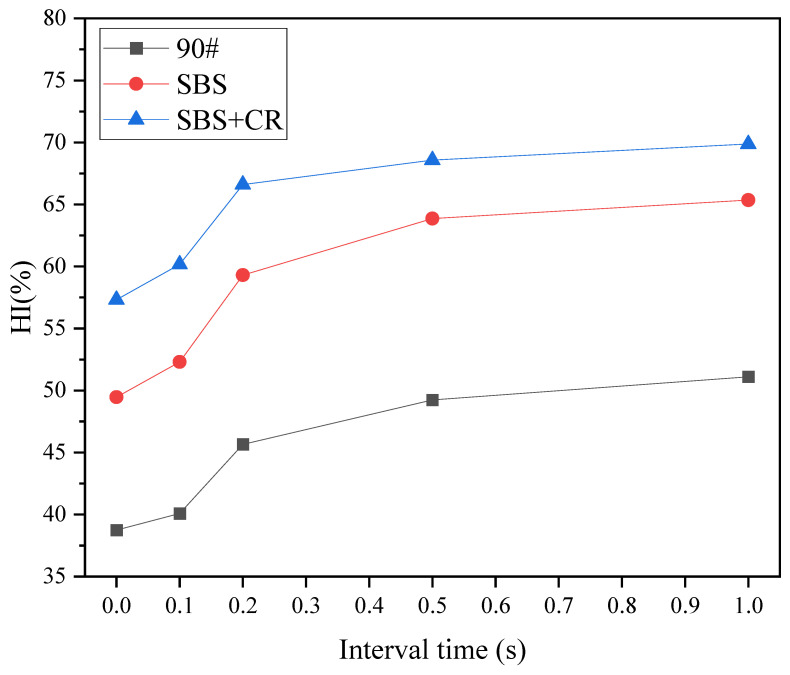
The change curve of fatigue–healing index with interval time.

The fatigue life of the three kinds of asphalt mixtures under the loading interval is obtained above, and the fatigue loading times corresponding to different degrees of damage can be obtained through the fatigue life. The test conditions were set as a healing temperature of 60 °C, healing time of 6 h, and fatigue damage of 50%. The test results are shown in Figure 10.

It can be seen from Figure 10 that the fatigue–healing index of different kinds of asphalt mixtures has the same change trend with the interval time, indicating that the type of asphalt does not affect the trend of fatigue–healing index change with different interval times. The fatigue–healing HI values of the three asphalt mixtures at the same interval time are determined by the fatigue properties of the asphalt itself. As the interval time becomes longer, the fatigue–healing HI value becomes larger, indicating that the healing effect will be better if the interval time is long enough. However, when the interval was 0.5 s and 1 s, the healing index increased slowly with time. It shows that there is the most cost-effective interval time. If the time is exceeded, the growth of the HI value of the asphalt mixture tends to be flat, and the improvement of healing ability is not apparent by extending the interval. It shows that controlling the driving speed on a highway can enhance the healing performance of asphalt pavement, but it needs to be controlled to an appropriate speed. This also confirms the research results of some scholars [28,29]. Speeds that are too slow have no significant effect on the fatigue–healing performance of the pavement.

## 4. Gray Correlation Analysis

Many factors affect the fatigue–healing performance of the asphalt mixture, such as the type, healing temperature, fatigue degree, loading interval, etc. However, researchers are more concerned about which influencing factor has the highest degree of influence [30]. This paper uses the gray correlation analysis method to analyze the correlation degree of each influencing factor.

Gray system theory regards all objective systems as integral and orderly and believes that the data in the system must also hide internal laws. The method of gray relational analysis is based on gray system theory by comparing the similarity of the geometric shapes determined by the reference data column and the comparison data column and judging whether the relationship between the two is close to reflect the degree of correlation between the curves. Its analysis steps are:

(1) Determine the data column (feature sequence and parent sequence), perform dimensionless (initialization or mechanization) processing on the data, and calculate the difference sequence;
(2) Solve the gray correlation coefficient between the parent sequence and the feature sequence;
(3) Solve and sort the gray relational values to conclude.

**Figure 11:**
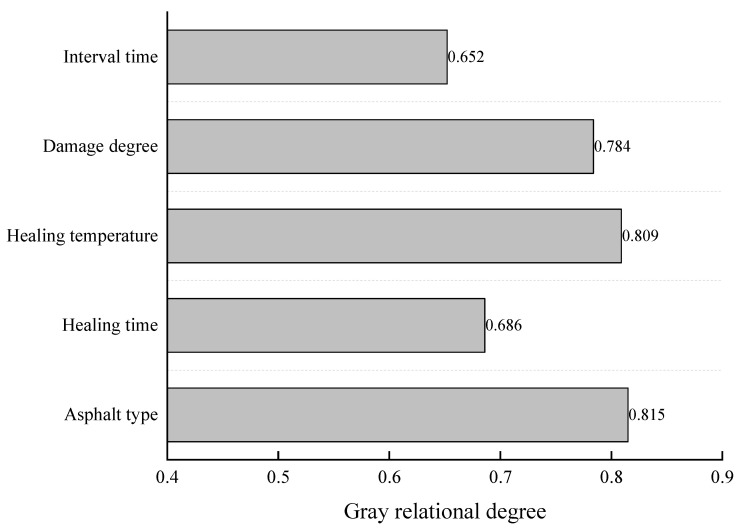
Gray correlation degree of each influencing factor.

In this study, the healing index HI was used as the parent sequence, and the gray correlation analysis was carried out on the five influencing factors of asphalt type, healing time, healing temperature, degree of damage and interval time. First, dimensionless processing of the experimental data is carried out. The interval time data contains many 0 s, and it cannot be initialized well, so this paper uses mean value processing data; secondly, calculate the difference sequence of each influencing factor; Finally, according to the principle of most minor information, take the resolution coefficient ρ = 0.5, and solve the gray correlation coefficient of each factor. Studies have shown that the fatigue performance of the asphalt mixture is closely related to its hardness [27,31], but the type of asphalt is a fixed variable. It cannot be calculated numerically, so the softening point (numerical value) is used instead. Table 3 shows the healing index processing results of the asphalt mixture fatigue test under different influencing factors. The analysis results are shown in Figure 11.

It can be seen from Figure 11 that the impact of various influencing factors on the healing index HI of asphalt mixture is as follows: asphalt type, healing temperature, degree of damage healing time, and interval time. According to the gray system theory, when the resolution coefficient ρ = 0.5, and if the gray correlation degree is more significant than 0.6, it indicates that the correlation degree is significant. Therefore, these five influencing factors are significantly related to the healing index of the asphalt mixture, and the type of asphalt has the most significant effect on the healing of the asphalt mixture.

## 5. Conclusions

In this study, F-H-F tests were carried out on three kinds of asphalt mixtures by semicircular bending test. The gray relational analysis test data is used to evaluate the effects of different types of asphalt, healing time, healing temperature, degree of damage and interval time on the fatigue–healing performance of asphalt mixture. The main conclusions drawn are as follows:

(1) The asphalt mixture has an optimal healing temperature, which is lower than the softening point. When the healing temperature is higher than the optimal healing temperature, the healing performance of the asphalt mixture is not improved.
(2) The healing index increased with the increase in healing time, but the growth rate slowed down after 6 h. There is a most cost-effective healing time, continuing to extend the healing time to improve the healing ability is not obvious.
(3) When the damage level was 10%, the HI value of the SBS-modified asphalt mixture and SBS + CR composite modified asphalt mixture reached 100.6% and 153.2%, indicating that the fatigue life after healing was higher than that under normal conditions. The “forging effect” occurs. The healing index of asphalt mixture increases with the increase in damage degree.
(4) The fatigue life of the asphalt mixture can be significantly improved by the interval time. The intermittent time of 0.1 s can increase the fatigue life by up to 38.84%. The effect of intermittent loading time on the fatigue life of asphalt mixture mainly occurs in the first 0.2 s after the load is removed, and has little effect on the fatigue life after 0.2 s.
(5) According to the test data of gray correlation analysis, the value of the gray correlation degree of asphalt type is the largest, which is 0.815. That is, the type of asphalt largely determines the fatigue–healing performance of the asphalt mixture. Other influencing factors in order of importance were: healing temperature, degree of damage, healing time and interval time.

The results did not consider the fatigue self-healing properties of reclaimed asphalt mixture. The healing properties of recycled asphalt mixture after fatigue loading will be studied in the future. In addition, more comprehensive performance tests will be conducted to assess the healing properties of the asphalt mixture in terms of resistance to rutting and freeze–thaw damage.

## Figures and Tables

**Table 1:** Basic indicators of asphalt.

Test Project	90# base asphalt	Specification Requirement	SBS	SBS + CR	Specification Requirement
Penetration (25 °C)/0.1 mm	89	80–100	68	64	60–80
Elongation (5 °C)/cm	100 (15 °C)	≥100	35.5 (5 °C)	38.1(5 °C)	≥30
Softening Point/°C	46	≥45	63	72	≥55
Viscosity (135 °C)/(Pa·s)	1.60	≤3	2.14	2.35	≤3

**Table 2:** Design gradation of asphalt mixture.

Sieve size (mm)	19.0	16.0	13.2	9.5	4.75	2.36	1.18	0.6	0.3	0.15	0.075
Passing rate (%)	100.0	95.0	84.0	70.0	48.0	34.0	24.5	17.5	12.5	9.5	6.0

**Table 3:** Influencing factors and healing index of asphalt mixture fatigue–healing.

Test Number	Softening Point (°C)	Healing Time (h)	Healing Temperature (°C)	Degree of Damage (%)	Interval Time (s)	HI (%)
1	46	6	60	50	0	38.7
2	63	6	60	50	0	49.5
3	72	6	60	50	0	57.3
……	……	……	……	……	……	……
47	63	6	60	50	1	65.4
48	72	6	60	50	1	69.9

## Data Availability

Not applicable.
